# The NESH/Abi-3-based WAVE2 complex is functionally distinct from the Abi-1-based WAVE2 complex

**DOI:** 10.1186/s12964-015-0119-5

**Published:** 2015-10-01

**Authors:** Saki Sekino, Yuriko Kashiwagi, Hitoshi Kanazawa, Kazuki Takada, Takashi Baba, Seiichi Sato, Hiroki Inoue, Masaki Kojima, Katsuko Tani

**Affiliations:** School of Life Sciences, Tokyo University of Pharmacy and Life Sciences, Hachioji, Tokyo 192-0392 Japan

**Keywords:** c-Abl, Abi-1, NESH/Abi-3, WAVE2, Imatinib mesylate, Membrane protrusions

## Abstract

**Background:**

Abl interactor (Abi) family proteins play significant roles in actin cytoskeleton organization through participation in the WAVE complex. Mammals possess three Abi proteins: Abi-1, Abi-2, and NESH/Abi-3. Abi-1 and Abi-2 were originally identified as Abl tyrosine kinase-binding proteins. It has been disclosed that Abi-1 acts as a bridge between c-Abl and WAVE2, and c-Abl-mediated WAVE2 phosphorylation promotes actin remodeling. We showed previously that NESH/Abi-3 is present in the WAVE2 complex, but neither binds to c-Abl nor promotes c-Abl-mediated phosphorylation of WAVE2.

**Results:**

In this study, we characterized NESH/Abi-3 in more detail, and compared its properties with those of Abi-1 and Abi-2. NESH/Abi-3 was ectopically expressed in NIH3T3 cells, in which Abi-1, but not NESH/Abi-3, is expressed. The expression of NESH/Abi-3 caused degradation of endogenous Abi-1, which led to the formation of a NESH/Abi-3-based WAVE2 complex. When these cells were plated on fibronectin-coated dishes, the translocation of WAVE2 to the plasma membrane was significantly reduced and the formation of peripheral lamellipodial structures was disturbed, suggesting that the NESH/Abi-3-based WAVE2 complex was unable to help produce lamellipodial protrusions. Next, Abi-1, Abi-2, or NESH/Abi-3 was expressed in v-*src*-transformed NIH3T3 cells. Only in NESH/Abi-3-expressed cells did treatment with an Abl kinase inhibitor, imatinib mesylate, or siRNA-mediated knockdown of c-Abl promote the formation of invadopodia, which are ventral membrane protrusions with extracellular matrix degradation activity. Structural studies showed that a linker region between the proline-rich regions and the Src homology 3 (SH3) domain of Abi-1 is crucial for its interaction with c-Abl and c-Abl-mediated phosphorylation of WAVE2.

**Conclusions:**

The NESH/Abi-3-based WAVE2 complex is functionally distinct from the Abi-1-based one, and NESH/Abi-3 may be involved in the formation of ventral protrusions under certain conditions.

**Electronic supplementary material:**

The online version of this article (doi:10.1186/s12964-015-0119-5) contains supplementary material, which is available to authorized users.

## Background

Abl interactor (Abi) family proteins are known to be involved in regulation of the actin cytoskeleton. Mammals possess three Abi proteins (Abi-1, Abi-2, and NESH/Abi-3). The Abi-1 and Abi-2 proteins were first identified on yeast two-hybrid screening for proteins that interact with a non-receptor tyrosine kinase, c-Abl [1, 2], which is the proto-oncogene product of the Abelson murine leukemia virus oncogene *v-abl* [3]. NESH/Abi-3 was identified as a new human gene that possesses a Src homology 3 (SH3) domain [4], and was later added to the Abi family based on the amino acid sequence similarity [5]. The three Abi proteins possess substantially the same domain structure, including an N-terminal WAVE-binding (WAB) domain, several proline-rich regions, poly-proline structures, and a C-terminal SH3 domain [5]. We and other groups previously showed that Abi-1 promotes the c-Abl-mediated phosphorylation of certain proteins such as Mena [6], BCAP [7], Cdc2 [8], and WAVE2 [9], suggesting its role in the regulation of Abl-mediated signal transduction. The regulation of c-Abl kinase activity by Abi-1-derived phosphopeptides was also reported by Xiong et al. [10]. Other studies showed that the Abi family proteins are critical regulators of actin dynamics [11]. Abi-1 and Abi-2, in particular, are present in a macromolecular WAVE complex, which regulates Arp2/3-mediated actin filament nucleation and actin network assembly in response to Rac GTPase [12–15]. The WAVE complex is composed of five proteins: WAVE, PIR121/Sra1, Nap1, HSPC300, and Abi. Mammals possess three WAVE proteins: WAVE1, 2, and 3. Binding studies indicated that Abi-1 directly interacts with WAVE2 and Nap1, and NAP1 interacts with PIR121/Sra1 [16]. Recent studies showed that Abi-1 connects c-Abl to WAVE2 to permit c-Abl-mediated WAVE2 phosphorylation. This promotes the activation of the WAVE2 complex leading to the formation of lamellipodial membrane protrusions [9]. Thus, among the five components, Abi-1 and possibly Abi-2 serve as mediators that couple c-Abl-mediated signal transduction and actin cytoskeleton reorganization.

Compared with those of Abi-1 and Abi-2, the function of NESH/Abi-3 remains mostly unclear. Ichigotani et al. reported that overexpression of NESH/Abi-3 in metastatic cells suppressed cellular motility and the metastasis potential [17]. Then, Matsuda et al. reported that NESH/Abi-3 expression enhanced metastasis in the presence of an Abl inhibitor, imatinib mesylate [18]. More recently, Bae et al. reported that NESH/Abi-3 directly binds to F-actin, and regulates dendritic spine morphogenesis and synapse formation in rat hippocampal neurons [19, 20]. These results indicate that NESH/Abi-3 is somehow involved in the regulation of the actin cytoskeleton, but the mechanism remains elusive. In addition, the similarities and differences among the three Abi family proteins have not been fully defined. In this context, we previously reported that NESH/Abi-3, like Abi-1 and Abi-2, is present in the WAVE2 complex, but neither binds to c-Abl nor promotes c-Abl-mediated phosphorylation of WAVE2 [21].

In this study, we further examined the function of NESH/Abi-3. Our results showed that the NESH/Abi-3-based WAVE2 complex is functionally distinct from the Abi-1-based one. We found another function of NESH/Abi-3, i.e., in the formation of membrane protrusions in v-Src-expressing cells.

## Results

### Ectopic expression of NESH/Abi-3 perturbed the formation of lamellipodial protrusions

**Fig. 1:**
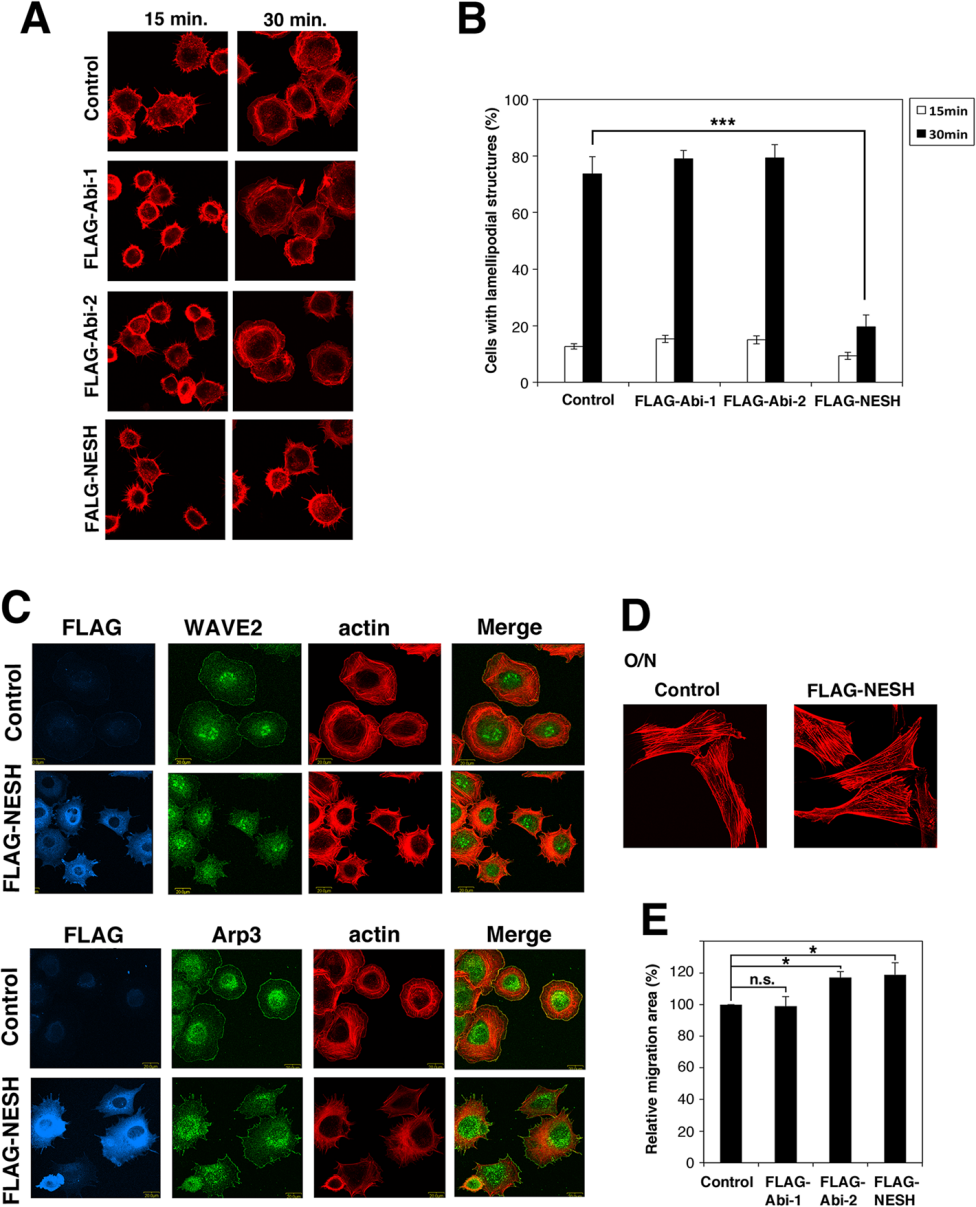
NESH/Abi-3 expression impaired WAVE2 membrane translocation and cell spreading. (**a**) Control NIH3T3 cells, and NIH3T3 cells expressing FLAG-Abi-1, FLAG-Abi-2, or FLAG-NESH were plated onto FN-coated coverslips. At the indicated times, the cells were fixed and stained with TRITC-phalloidin. (**b**) Quantitative analysis of cells in (**a**). Cells with lamellipodial structures were counted under a fluorescence microscope. At least 100 cells were analyzed for each sample. Data represent the means ± S.D. for three independent experiments. Error bars represent S.D. ***, P < 0.001, Student's *t* test. (**c**) The control and FLAG-NESH-expressing NIH3T3 cells were plated onto FN-coated coverslips. One hour after plating, the cells were fixed and stained with an anti-FLAG antibody, TRITC-phalloidin, an anti-WAVE2, or an anti-Arp3 antibody. The right panels show merged views of the TRITC-phalloidin and WAVE2 or Arp3 stained images. (**d**) The control and FLAG-NESH-expressing NIH3T3 cells were plated onto FN-coated coverslips. After overnight incubation, the cells were fixed and stained with TRITC-phalloidin. (**e**) The wound-healing assay was performed, and the migration area was quantified as described under Materials and Methods. Data represent the means ± S.D. for three independent experiments. The value for the control cells was set to 100 %. Error bars represent S.D. *, P < 0.05; n.s., the difference was not significant, Student's *t* test

To identify a function of NESH/Abi-3, we first focused on the WAVE2 complex because our previous study showed that NESH/Abi-3 is included in the complex [21]. The importance of the linkage between Abi-1 and WAVE2 in the formation of lamellipodial protrusions has been reported [9, 22]. The level of expression of NESH/Abi-3 is very low in NIH3T3 cells (Additional file 1: Figure S1a). Accordingly, FLAG-tagged NESH/Abi-3 was stably expressed in NIH3T3 cells using a recombinant retrovirus and then cell spreading on a fibronectin (FN)-coated plate was examined (Fig. 1a). At 15 min after the plating, both control and NESH/Abi-3-expressing NIH3T3 cells were attached to the dishes and filopodial membrane protrusions were observed. At 30 min, control cells had lamellipodial membrane protrusions around their periphery and were well spread on the dish (Fig. 1a, top right image). By contrast, the NESH/Abi-3-expressing cells did not exhibit lamellipodial protrusions and were poorly spread on the dish at 30 min. (bottom right image). FLAG-tagged Abi-1- or Abi-2-expressing cells exhibited a cell spreading pattern similar to that of the control cells (second and third row images), suggesting that the effect was specific to NESH/Abi-3. Quantitative analysis supported this viewpoint (Fig. 1b). Rac GTPase regulates the formation of lamellipodial structures [23]. We then examined Rac activity in the NESH/Abi-3-expressing cells. The CRIB domain, which specifically binds to activated Rac (i.e., a GTP-binding form), was used to precipitate the activated Rac from cell lysates. As shown in Additional file 1: Figure S1f, a significant amount of activated Rac was detected at 15 min after plating for both control and NESH/Abi-3-expressing NIH3T3 cells. No significant differences were observed between the two types of cells, suggesting that the signaling pathway leading to Rac activation may not be disturbed upon the expression of NESH/Abi-3. Leng et al. reported that WAVE2 and c-Abl were translocated to the cell periphery upon stimulation by FN, and that their locations are important for the formation of lamellipodial protrusions and cell spreading [9]. We thus examined the localization of endogenous WAVE2 in the control and NESH/Abi-3-expressing NIH3T3 cells after plating. As shown in Fig. 1c, WAVE 2 was localized to the plasma membrane of the control cells (top row images), but not that of the NESH/Abi-3-expressing cells (second row images). Meanwhile, Arp3 was localized to the cell periphery in both the control and NESH/Abi-3-expressing NIH3T3 cells (third and bottom row). These results suggest that WAVE2 was not properly localized to the cell periphery in the NESH/Abi-3-expressing cells after plating.

The actin cytoskeleton in NESH/Abi-3-expressing NIH3T3 cells looked normal overnight after plating (Fig. 1d). We then examined the lateral motility of cells by using a wound healing assay (Fig. 1e). Here, the expression of NESH/Abi-3 did not inhibit migration of NIH 3T3 cells. This result differed from those obtained using v-*src*-tranformed NIH3T3 cells, in which the expression of NESH/Abi-3 inhibits cell motility (17, Fig. 4b). We discuss the difference in the Discussion section. To exclude the possibility that the FLAG-tag affected the results, we obtained NIH3T3 cells expressing Abi-1 or NESH/Abi-3 without any tag. In terms of the formation of the lamellipodial structures after plating and the lateral cell motility, similar results were obtained using the non-tagged proteins (Additional file 1: Figure S1b–d).

### NESH/Abi-3-based WAVE2 complex

**Fig. 2:**
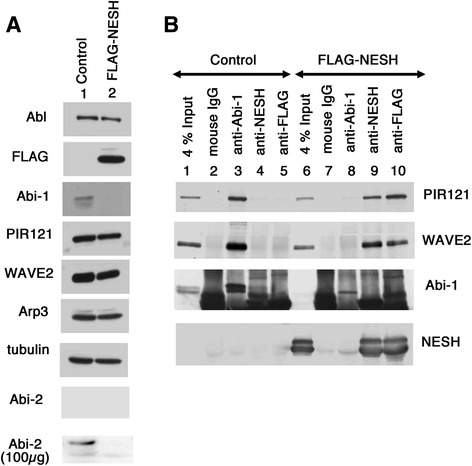
Ectopically expressed NESH/Abi-3 was incorporated into the WAVE2 complex. (**a**) Twenty μg aliquots of lysates prepared from the control and FLAG-NESH-expressing cells were analyzed by Western blotting with the indicated antibodies. For Abi-2, data regarding 100 μg aliquots of lysates are also shown because no signal was found when 20 μg aliquots were used. (**b**) The lysates of the control and FLAG-NESH-expressing cells were immunoprecipitated with mouse IgG, an anti-Abi-1 (1G9), an anti-NESH (2H7), or an anti-FLAG (M2) antibody. The precipitated proteins were analyzed by Western blotting with the indicated antibodies. To confirm the NESH precipitation, a rabbit anti-human NESH antibody was also used

The above results prompted us to examine the WAVE2 complex in NESH/Abi-3-expressing cells. We first checked endogenous expression of its components: WAVE2, Abi-1, and PIR121/Sra1 (Fig. 2a). These proteins were detected in the control cells (left column). In the NESH/Abi-3-expressing NIH3T3 cells, the expression levels of WAVE2 and PIR121/Sra1 were verified, but the amount of Abi-1 protein was markedly decreased compared with that in the control cells. The amount of Abi-2 protein also decreased in NESH/Abi-3-expressing cells (Fig. 2a, bottom row). Because the expression level of Abi-2 was relatively low in NIH3T3 cells (Additional file 1: Fig. S1a), we focused on Abi-1 and NESH/Abi-3 in NIH3T3 cells. To investigate the WAVE2 complex in the control and NESH/Abi-3-expressing NIH3T3 cells, we immunoprecipitated Abi-1 or NESH/Abi-3 (Fig. 2b). Abi-1 was precipitated using an anti-Abi-1 antibody from the control and NESH/Abi-3-expressing NIH3T3 cell lysates (lanes 3 and 8). As expected, little Abi-1 was precipitated from the NESH/Abi-3-expressing NIH3T3 cell lysates (lane 8). WAVE2 and PIR121/Sra1 were co-precipitated with Abi-1 from the control cell lysates (lane 3, first and second rows), and their precipitation was markedly reduced when the NESH/Abi-3-expressing NIH3T3 cell lysates were used (lane 8, first and second rows). NESH/Abi-3 was precipitated with an anti-NESH/Abi-3 antibody (lanes 4 and 9) or an anti-FLAG antibody (lanes 5 and 10). Both WAVE2 and PIR121/Sra1 were co-precipitated with NESH/Abi-3 from the NESH/Abi-3-expressing NIH3T3 cell lysates. A control mouse IgG was not precipitated with any of PIR121/Sra1, WAVE2, Abi-1, and NESH/Abi-3 from either cell lysates (lanes 2 and 7). As mentioned above, Abi-1 directly binds to WAVE2, but not to PIR121/Sra1, so the precipitation of PIR121/Sra1 with an antibody against each Abi protein indicated the formation of a WAVE2 complex. Thus, the results suggest the formation of a NESH/Abi-3-based WAVE2 complex in the NESH/Abi-3-expressing NIH3T3 cells.

**Fig. 3:**
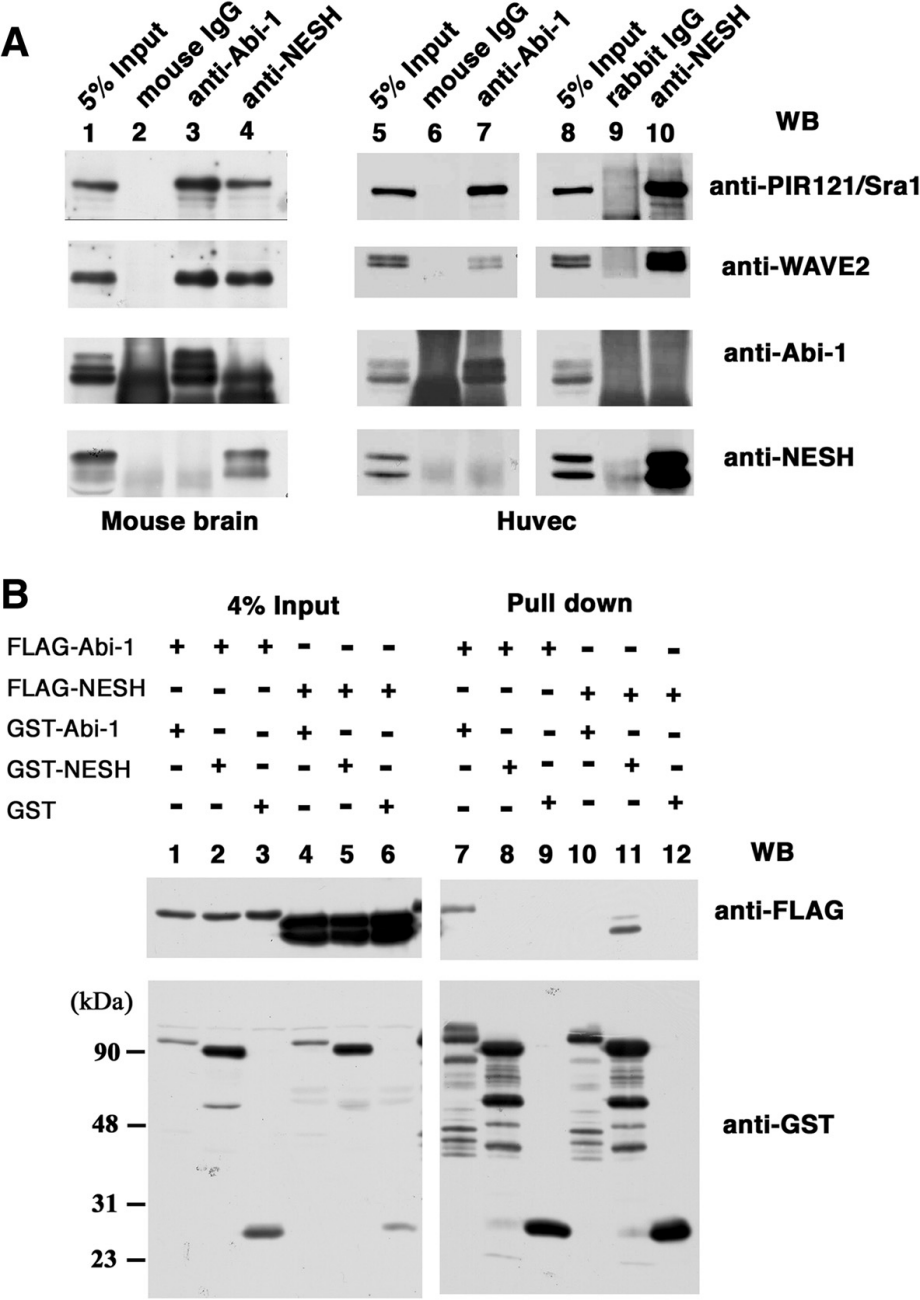
Abi-1-based and NESH/Abi-3-based WAVE2 complexes exist independently. (**a**) Lysates prepared from mouse brain were immunoprecipitated with a mouse IgG, an anti-Abi-1 monoclonal antibody (1G9), or an anti-NESH monoclonal antibody (2H7) (lanes 2 to 4). Lysates prepared from HUVEC were immunoprecipitated with the mouse IgG, the anti-Abi-1 monoclonal antibody (1G9), a rabbit IgG, or a rabbit anti-human NESH antibody as indicated (lanes 6, 7, 9, and 10). The precipitated proteins were analyzed by Western blotting with the indicated antibodies. A rabbit anti-mouse NESH antibody was used to detect NESH in the mouse brain lysates (lanes 1 to 4). The rabbit (lanes 5 to 7) or mouse (lanes 8 to 10) anti-human NESH antibody was used to detect NESH in the HUVEC lysates. (**b**) FLAG-Abi-1 or FLAG-NESH was coexpressed with GST-Abi-1, GST-NESH, or GST in 293 T cells. GST-Abi constructs were pulled down with glutathione beads, and then the bound proteins were detected by Western blotting with an anti-FLAG or GST antibody (pull-down). To determine the amounts of the proteins expressed, 4 % of each lysate was analyzed (4 % Input)

To clarify whether or not the Abi-1-based and NESH/Abi-3-based WAVE2 complexes exist independently, we examined mouse brain and HUVEC lysates, each containing significant amounts of NESH/Abi-3 as well as Abi-1. We immunoprecipitated Abi-1 or NESH/Abi-3 by using specific antibodies (Fig. 3a). From mouse brain lysates, WAVE2 and PIR121/Sra1 were co-precipitated with Abi-1 and NESH/Abi-3 (lanes 3 and 4). When an anti-Abi-1 antibody was used, no NESH/Abi-3 was co-precipitated (lane 3, fourth row). However, it was not clear whether or not Abi-1 was co-precipitated with an anti-NESH/Abi-3 antibody (lane 4, third row). A thick smear band was detected near the position of Abi-1 on the blot. This was partly because the anti-NESH/Abi-3 mouse monoclonal antibody used for the precipitation was detected with an anti-mouse secondary antibody. A control mouse IgG did not precipitate PIR121/Sra1, WAVE2, Abi-1, or NESH/Abi-3 (lane 2). For HUVEC cells, we used an anti-NESH/Abi-3 rabbit polyclonal antibody for the immunoprecipitation, this antibody reacting with human NESH/Abi-3 (Fig. 3a, right panels). In the HUVEC cells, both Abi-1 and NESH/Abi-3 co-precipitated WAVE2 and PIR121/Sra1 (lanes 7 and 10). At this time, co-precipitation of Abi-1 and NESH/Abi-3 was not detected using either the anti-Abi-1 antibody (lane 7) or the anti-NESH antibody (lane 10). This clearly indicated that the NESH/Abi-3-based WAVE2 complex was independent of the Abi-1-based one. The interaction of c-Abl with the WAVE complex was reported by others [9, 22]. We then examined the binding of c-Abl to either the Abi-1-based or NESH/Abi-3-based WAVE2 complex. However, we were unable to detect the co-precipitation of c-Abl with the anti-Abi-1 or anti-NESH/Abi-3 antibody from brain lysates (Additional file 1: Figure S1e), suggesting that the interaction might be transient and/or unstable.

Meanwhile, Fan et al. reported the oligomeric character of Abi-1 [24]. To determine whether or not Abi-1 interacts with NESH/Abi-3, we examined 293 T cells expressing both proteins. Abi-1 and NESH/Abi-3 were tagged with either a FLAG or GST. The GST-tagged proteins were precipitated with glutathione beads and co-precipitation was assessed with the anti-FLAG antibody (Fig. 3b). FLAG-Abi-1 was co-precipitated with GST-Abi-1 (lane 7), suggesting oligomerization of Abi-1. No FLAG-Abi-1 was co-precipitated with GST-NESH/Abi-3 (lane 8). Likewise, FLAG-NESH/Abi-3 was co-precipitated with GST-NESH/Abi-3 (lane 11), but not with GST-Abi-1 (lane 10). GST was precipitated with neither FLAG-Abi-1 (lane 9) nor FLAG-NESH/Abi-3 (lane 12). These results indicated that Abi-1 and NESH/Abi-3 are independent of each other as to the formation of an oligomer, supporting the idea that Abi-1 and NESH/Abi-3 create distinct WAVE2 complexes. Collectively, our results suggest that the NESH/Abi-3-based WAVE2 complex, which was produced in the NESH/Abi-3 expressing cells, cannot be localized to the cell periphery upon FN stimulation in order to produce the lamellipodial protrusions.

### NESH/Abi-3 promoted invadopodia formation in an imatinib-dependent manner

**Fig. 4:**
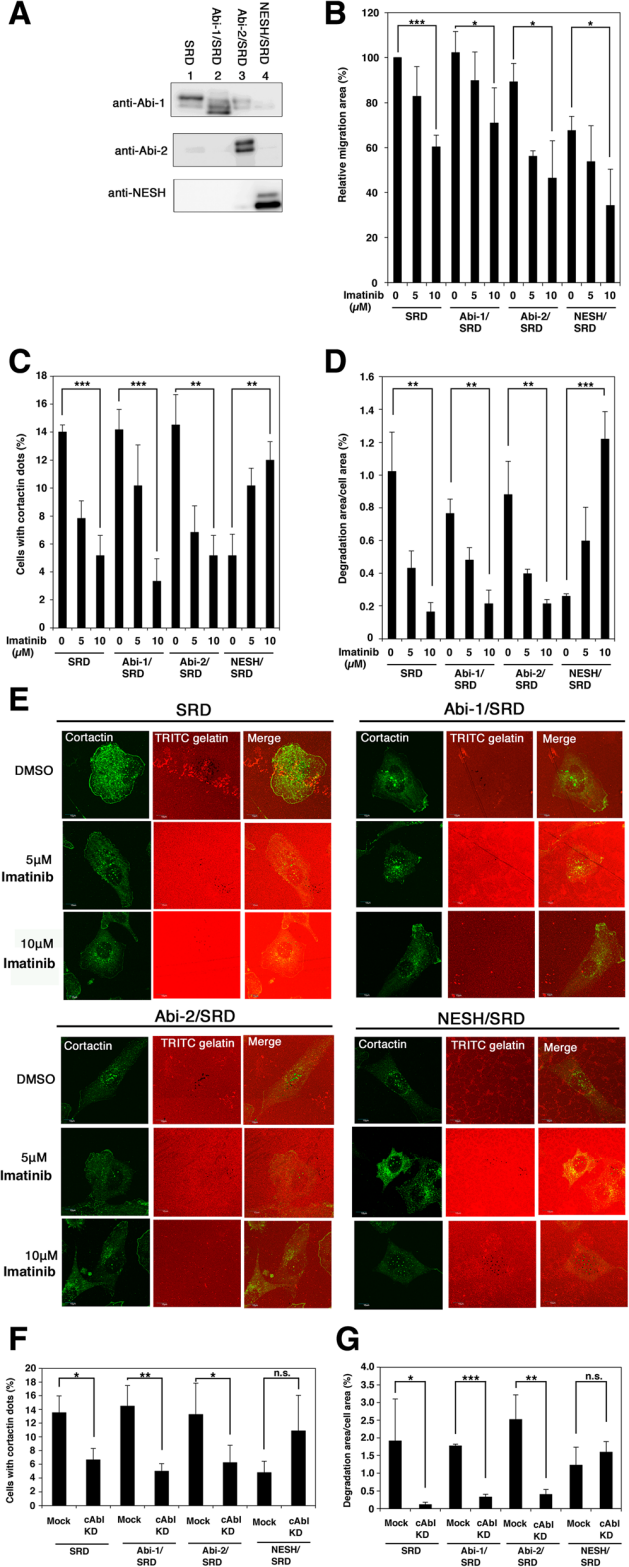
NESH/Abi-3 promoted invadopodia formation in the presence of imatinib. (**a**) Lysates were prepared from SRD cells, and SRD cells expressing Abi-1, FLAG-Abi-2, or NESH. Western blotting analysis was performed with the indicated antibodies. (**b**) The wound-healing assay was performed, and the migration area was quantified as described under Materials and Methods. Data represent the means ± S.D. for three independent experiments. Error bars represent S.D. The value for the control SRD cells was set to 100 %. *, *P* < 0.05; ***, *P* < 0.001, Student's *t* test. (**c**) The indicated cells were plated onto TRITC gelatin-coated dishes. After 7 h, cells were fixed and stained with an anti-cortactin antibody. Cells with a punctate cortactin-rich structure were counted under a fluorescence microscope. At least 100 cells were analyzed for each sample. Data represent the means ± S.D. for three independent experiments. Error bars represent S.D. **, *P* < 0.005; ***, *P* < 0.001, Student's *t* test. (**d**) Invadopodia degradation areas were quantified as described under Materials and Methods. For each sample, five cells exhibiting invadopodia degradation were randomly picked up and the degradation area/cell area was calculated. Data represent the means ± S.D. for three independent experiments. Error bars represent S.D. **, P < 0.005; ***, *P* < 0.001, Student's *t* test. (**e**) Representative confocal microscopy images of SRD cells, and SRD cells expressing Abi-1, FLAG-Abi-2, or NESH in (D). The black dots indicate the invadopodia-mediated gelatin degradation. (**f**, **g**) SRD cells and SRD cells expressing Abi-1, FLAG-Abi-2, or NESH were treated with the c-Abl siRNA for 72 h, and then analyzed as described in (**c**) and (**d**). (**f**) The percentage of cells containing a punctate cortactin-rich structure. (**g**) Quantification of the degradation area. Data represent the means ± S.D. for three independent experiments. Error bars represent S.D. *, *P* < 0.05; **, *P* < 0.005; ***, *P* < 0.001; n.s., the difference was not significant (*P* = 0.062 in (**f**), *P* = 0.17 in (**g**)), Student's *t* test

Although Abi-1 promotes c-Abl-mediated phosphorylation, NESH/Abi-3 does not [21]. Hence, Abi-1 and NESH/Abi-3 may have different functions in the c-Abl-mediated signaling pathway. To examine the function of NESH/Abi-3, we next used an Abl kinase inhibitor (imatinib mesylate, Gleevec) to treat v-*src*-transformed NIH3T3 cells (SRD cells). This is because Matsuda et al. reported that vascular metastasis, which was assessed by observing lung colonization after tail vein injection, was elevated with imatinib mesylate-treatment in NESH/Abi-3-expressing SRD cells [18]. We thus expressed the three Abi family proteins in the SRD cells so as to compare the phenotypes among the Abi family proteins. The expression of Abi-1, Abi-2, and NESH/Abi-3 in the SRD cells was first verified (Fig. 4a). Similar to in the case of NIH3T3 cells, the level of expression of the Abi-1 protein was remarkably reduced in NESH/Abi-3-expressing SRD cells (Fig. 4a, top row, lane 4). We next examined lateral cell motility by using a wound healing assay. As shown in Fig. 4b, SRD cells expressing NESH/Abi-3, but not Abi-1 or Abi-2, showed somewhat reduced motility compared with the parent SRD cells. Imatinib-treatment reduced the motility of the control SRD cells, and also that of Abi-1- Abi-2, or NESH/Abi-3-expressing SRD cells, suggesting that the imatinib-treatment similarly inhibited cellular lateral motility. We then examined invadopodia formation (Fig. 4c, d and e). Invadopodia are actin-rich ventral membrane protrusions, which possess extracellular matrix-degrading activity, and are supposed to be involved in cancer cell invasion [25]. Cortactin is a well-known marker for invadopodia. Invadopodium precursors are punctate, non-degrading cortactin-rich structures, whereas mature invadopodia are structures that can degrade the extracellular matrix [26]. SRD cells expressing Abi-1, Abi-2, or NESH/Abi-3 were seeded onto a plate coated with fluorescently labeled gelatin and then cultured. After 7 h, the cells were fixed and the number of cells exhibiting punctate cortactin staining was determined (Fig. 4c). In Fig. 4d and e, the gelatin degradation area was measured. When the control SRD cells were treated with increasing amounts of imatinib, the number of cells with cortactin dots as well as the degradation area decreased, suggesting that c-Abl-mediated phosphorylation is involved in the formation of invadopodia, as reported previously [27]. Abi-1- or Abi-2-expressing SRD cells showed substantially the same levels of invadopodia formation as the parent SRD cells, and the imatinib-treatment suppressed the invadopodia formation. In contrast to these two cases, the ectopic expression of NESH/Abi-3 suppressed the invadopodia formation. It should be noted that the imatinib-treatment remarkably increased the invadopodia formation of the NESH/Abi-3-expressing SRD cells. To determine whether or not the observed effects were definitely due to the inhibition of c-Abl kinase, we knocked down c-Abl in Abi-1- Abi-2, or NESH/Abi-3-expressing SRD cells and then analyzed the invadopodia formation. As shown in Fig. 4f and g, the knockdown of c-Abl suppressed the invadopodia formation by Abi-1- or Abi-2-expressing SRD cells. In addition, the treatment increased the invadopodia formation by the NESH/Abi-3-expressing SRD cells, although it was not marked when compared with in the case of imatinib-treatment. These results indicate that the expression of NESH/Abi-3, but not Abi-1 or Abi-2, increased invadopodia formation when c-Abl was inhibited, suggesting that NESH/Abi-3 has a different role from that of Abi-1 and Abi-2 in the c-Abl-mediated signaling pathway and the invadopodia formation.

### Chimeric analyses

**Fig. 5:**
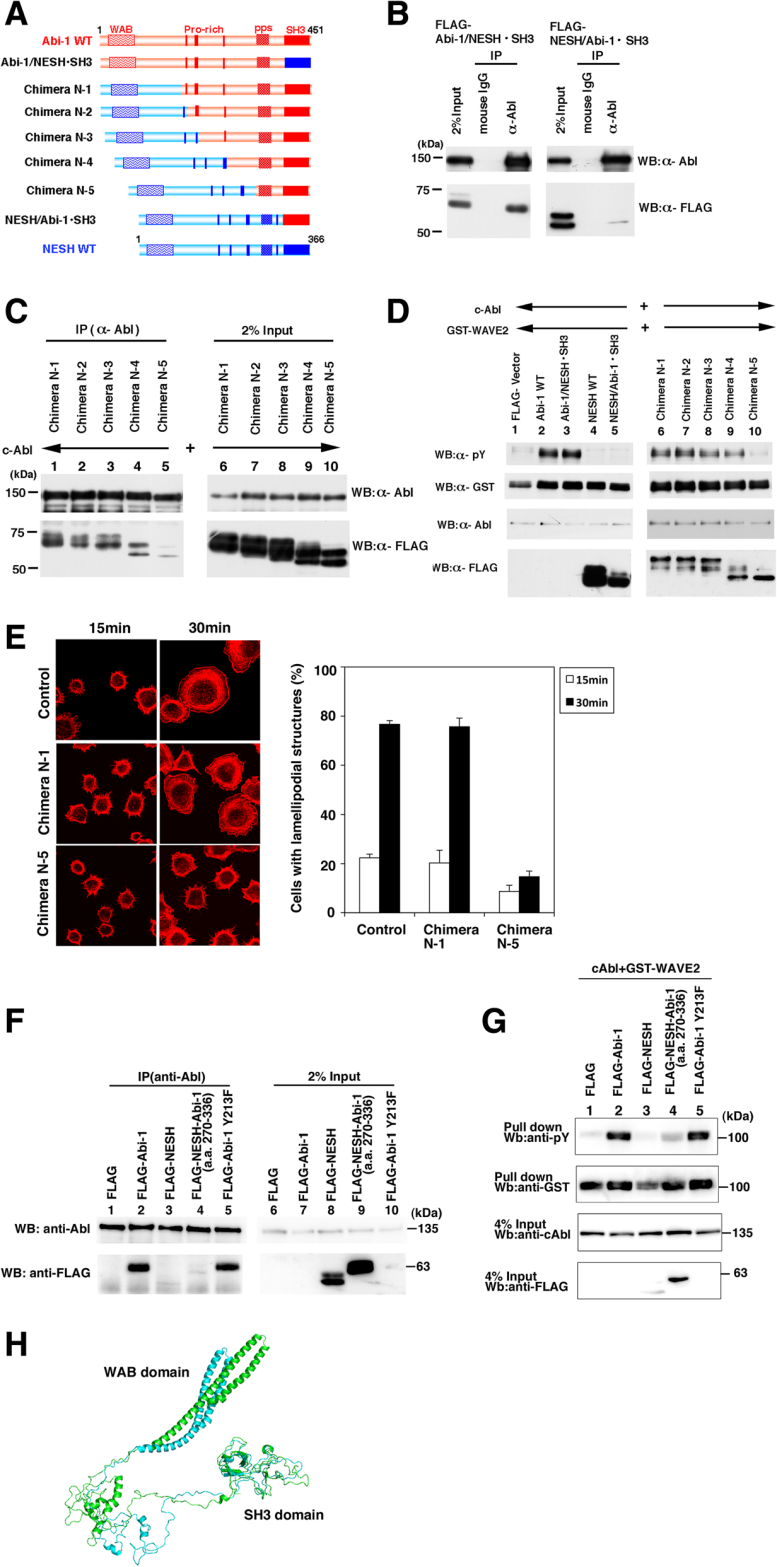
Analysis of Abi-1/NESH chimeras. (**a**) Schematic representation of the chimeric proteins. Abi-1 (red) and NESH (blue), and the chimeric proteins used in this study are schematically illustrated. The positions of the WAVE-binding (WAB) domain, the proline-rich region (PXXP motif), the polyproline structure (pps), and the SH3 domain are indicated. (**b**) FLAG-Abi-1/NESH•SH3 or FLAG-NESH/Abi-1•SH3 was coexpressed with c-Abl in 293T cells. A mouse IgG or an anti-c-Abl antibody was added to lysates of the transfected cells, and the precipitated proteins were analyzed by Western blotting with antibodies against c-Abl and FLAG. To determine the amounts of the proteins expressed, 2 % of each lysate was analyzed (2 % Input). (**c**) FLAG-chimera N-1 (lane 1), FLAG-chimera N-2 (lane 2), FLAG-chimera N-3 (lane 3), FLAG-chimera N-4 (lane 4), or FLAG-chimera N-5 (lane 5) was coexpressed with c-Abl in 293T cells. c-Abl was immunoprecipitated with the anti-c-Abl antibody, and the precipitated proteins were analyzed as described in (B). (**d**) 293T cells were cotransfected with expression plasmids for c-Abl and GST-WAVE2, together with an expression plasmid for FLAG (lane 1), FLAG-Abi-1 wild-type (lane 2), FLAG–Abi-1/NESH•SH3 (lane 3), FLAG-NESH wild-type (lane 4), FLAG–NESH/Abi-1•SH3 (lane 5), FLAG-chimera N-1 (lane 6), FLAG-chimera N-2 (lane 7), FLAG-chimera N-3 (lane 8), FLAG-chimera N-4 (lane 9), or FLAG-chimera N-5 (lane 10). The resulting cell lysates were analyzed by Western blotting with each of the antibodies against phosphotyrosine (α-pY), c-Abl, FLAG, and GST. The expression of the wild-type Abi-1 and Abi-1/NESH•SH3 was below the respective detection limit in this assay. However, the phosphorylation of GST-WAVE2 was evident in these cell lysates. The phosphorylated Abi-1 may have been subjected to degradation. (**e**) NIH3T3 cells expressing Chimera N-1 or Chimera N-5 were plated onto FN-coated coverslips. At the indicated times, the cells were fixed and stained with TRITC-phalloidin (left panels). Cells with lamellipodial structures were counted under a fluorescence microscope. At least 100 cells were analyzed for each sample. Data represent the means ± S.D. for three independent experiments. Error bars represent S.D. (the right graph) (**f**) FLAG (lane 1), FLAG-Abi-1 (lane 2), FLAG-NESH (lane 3), FLAG-NESH-Abi-1(a.a. 270–336) (lane 4), or FLAG-Abi-1 Y213F (lane 5) was coexpressed with c-Abl in 293T cells. c-Abl was immunoprecipitated with an anti-c-Abl antibody, and the precipitated proteins were analyzed as described in (**b**). To determine the amounts of the proteins expressed, 2 % of each lysate was analyzed (2 % Input). The expression levels of FLAG-Abi-1 and FLAG-Abi-1 Y213F were low compared with those of FLAG-NESH and FLAG-NESH-Abi-1(a.a. 270–336). However, the precipitation of FLAG-Abi-1 and FLAG-Abi-1 Y213F was evident in these cell lysates. (**g**) 293T cells were cotransfected with expression plasmids for c-Abl and GST-WAVE2, and together with an expression plasmid for FLAG (lane 1), FLAG-Abi-1 (lane 2), FLAG-NESH (lane 3), FLAG-NESH-Abi-1(a.a. 270–336) (lane 4), or FLAG-Abi-1 Y213F (lane 5). The GST-WAVE2 was pulled down with glutathione beads and analyzed by Western blotting with the antibodies against a phosphotyrosine and GST. To determine the amounts of proteins expressed, 4 % of each lysate was analyzed by Western blotting with the antibodies against c-Abl and FLAG (4 % Input). The expression of FLAG-Abi-1 and FLAG-Abi-1 Y213F was below the detection limit in this assay. However, the phosphorylation of GST-WAVE2 was evident in these cell lysates. (**h**) The predicted structures of Abi-1 and NESH. The structures of Abi-1 (green) and NESH (cyan) are presented as a ribbon diagram, being maximally superimposed with respect to their SH3 domains

To elucidate the structural difference between Abi-1 and NESH/Abi-3, we created a series of chimeric mutants between Abi-1 and NESH/Abi-3 (Fig. 5a). The activity of c-Abl is normally suppressed through an autoinhibitory mechanism [28], and interaction with partner proteins is involved in the regulation of its activity [29]. Dai and Pendergast [2] used a series of deletion mutants to analyze Abi-2 regions that are involved in the interaction with c-Abl. Their results revealed that the most N-terminal proline-rich region and the SH3 domain of Abi-2 are both involved in the interaction with c-Abl. We first examined whether or not the intrinsic SH3 domain of Abi-1 is essential for the binding to c-Abl. Expression plasmids for c-Abl and FLAG-tagged chimeras were cotransfected into 293 T cells. The cell lysates were subjected to immunoprecipitation using an anti-c-Abl antibody (Fig. 5b). The results demonstrated that c-Abl was co-precipitated with the Abi-1/NESH**•**SH3 mutant but not with the NESH/Abi-1**•**SH3 one. This finding showed that the SH3 domain of Abi-1 was functionally replaced by the SH3 domain of NESH/Abi-3, and that the SH3 domain of Abi-1 alone was insufficient for the binding to c-Abl. Next, we used proline-rich-region-swapping mutants to determine which proline-rich regions are involved in the binding between c-Abl and Abi-1. Chimera N-1 contained the WAB domain of NESH/Abi-3, and Chimeras N-2, N-3, and N-4 included the most N-terminal, N-terminal two, and N-terminal three proline-rich regions derived from NESH/Abi-3, respectively (Fig. 5a). As shown in Fig. 5c (lanes 1–4), c-Abl co-precipitated Chimeras N-1 to N-4. However, the coprecipitation of Chimera N-5 was markedly reduced, suggesting the difference between Chimera N-4 and Chimera N-5 was significant with respect to their binding to c-Abl. The difference comprises truncation of Abi-1 amino acids 270 to 336. Unexpectedly, this region contains no proline-rich motif. To further verify the functional significance of the interaction, we examined Abi-1-induced WAVE2 phosphorylation by c-Abl. We coexpressed c-Abl, GST-WAVE2, and each of the mutants in 293 T cells, and then examined the tyrosine phosphorylation of GST-WAVE2 (Fig. 5d). Specifically, when c-Abl and GST-WAVE2 were coexpressed with the Abi-1 WT (lane 2) or Abi-1/NESH**•**SH3 mutant (lane 3), the tyrosine phosphorylation of GST-WAVE2 was observed. However, when c-Abl and GST-WAVE2 were coexpressed with the NESH/Abi-3 WT (lane 4) or NESH/Abi-1**•**SH3 mutant (lane 5), no tyrosine phosphorylation of GST-WAVE2 was observed. Coexpression of each of Chimeras N1 to N4 promoted the tyrosine phosphorylation of GST-WAVE2 (lanes 6 to 9), although Chimera N-4 induced a slightly reduced level of phosphorylation (lane 9). Interestingly, coexpression of Chimera N-5 did not induce the phosphorylation of GST-WAVE2 (lane 10). This pattern of phosphorylation is consistent with the pattern of binding between c-Abl and each of the mutants. We then examined the cellular spreading of NIH3T3 cells expressing Chimera N-1 or N-5. Thirty minutes after plating, cells expressing Chimera N-1, but not Chimera N-5, produced lamellipodial protrusions (Fig. 5e). This result also suggests that Chimera N-5 behaves like NESH/Abi-3.

These results suggest the importance of amino acids 270 to 336 of Abi-1. We thus created a NESH/Abi-3 mutant that possesses amino acids 270 to 336 of Abi-1. Amino acids 270 to 336 of Abi-1 were inserted immediately after the third proline-rich region of NESH/Abi-3, and the resulting mutant was examined for the binding to c-Abl and the promotion of c-Abl-mediated phosphorylation of WAVE2. As shown in Fig. 5f and g, the mutant very slightly bound to c-Abl, and partially rescued the phosphorylation of WAVE2 by c-Abl, suggesting the importance of this region. Figure 5h shows the superimposed structures of Abi-1 and NESH/Abi-3, the structures being constructed by computer modeling. The two structures were similar to each other in terms of the SH3 domain (residues 392 to 451 of Abi-1 and residues 311 to 366 of NESH/Abi-3). The root-mean-square deviation **(**RMSD) for Cα atoms in this domain was 0.10 Å. This is consistent with our results demonstrating that the SH3 domains of Abi-1 and NESH/Abi-3 are interchangeable. The modeling results also demonstrated that the structures of the WAB domains were quite similar in the two proteins, suggesting that the structural difference between Abi-1 and NESH/Abi-3 is mainly due to the region between the WAB domain and the SH3 domain. Interestingly, the distance between the centers of the most N-terminal proline-rich region and the SH3 domain was 84.5 Å for Abi-1 and 76.2 Å for NESH/Abi-3 in this model. We also examined another mutant in which tyrosine 213 of Abi-1 was replaced by a phenylalanine, because tyrosine 213 of Abi-1 is reportedly phosphorylated by c-Abl and by BCR-Abl [10, 30]. In our system, this mutant and Abi-1 both bound to c-Abl and promoted the phosphorylation of WAVE2 by c-Abl in substantially the same manner (Fig. 5f, lane 5, and Fig. 5g, lane 5). NESH/Abi-3 does not have a tyrosine residue corresponding to the tyrosine 213 of Abi-1. But the absence of the corresponding phosphorylation in NESH/Abi-3 alone may not account for why NESH/Abi-3 does not regulate c-Abl-mediated phosphorylation of WAVE2.

## Discussion

In this study, we examined the roles of NESH/Abi-3 in the formation of membrane protrusions, lamellipodia and invadopodia. Our results suggest that NESH/Abi-3 plays a distinct role in the regulation of the actin cytoskeleton reorganization when compared with Abi-1 and Abi-2.

Several studies showed that depletion of Abi-1 leads to degradation of the WAVE2 complex. For example, siRNA-mediated knockdown of Abi-1 caused degradation of WAVE2 and other complex components [13, 31]. In fibroblasts derived from Abi-1 knockout mice, the protein levels of WAVE2, PIR121/Sra1, and Nap1 were significantly decreased [32]. In this study, we showed that expression of NESH/Abi-3 in NIH3T3 cells caused a marked reduction in the protein level of endogenous Abi-1. Differing from in the case of Abi-1 knockdown or knockout, the protein levels of WAVE2 and PIR121/Sra1 did not change, and a WAVE2 complex containing NESH/Abi-3 was formed, suggesting NESH/Abi-3 took the place of Abi-1. Our immunoprecipitation study showed that Abi-1 and NESH/Abi-3 are mutually exclusive and create distinct WAVE2 complexes. Translocation of WAVE2 to the cell periphery was decreased in the NESH/Abi-3-expressing cells. Leng et al. reported the importance of the c-Abl-mediated WAVE2 phosphorylation for driving actin polymerization [9]. As reported previously, Abi-1, but not NESH/Abi-3, promoted the WAVE2 phosphorylation by c-Abl [21]. The lack of the WAVE2 phosphorylation may lead to a reduction in the formation of lamellipodial protrusions. To support this idea, Chimera N-5, which lacks the ability to promote the WAVE2 phosphorylation, showed reduced lamellipodial formation (Fig. 5e). Meanwhile, we tried to detect endogenous phosphorylation of WAVE2 in the control NIH3T3 cells, but failed. The phosphorylation may occur transiently or be very low in NIH3T3 cells. As another possibility, Abi-1, but not NESH/Abi-3, might serve as a scaffold for proteins that support the formation of membrane protrusions. It was reported that the interaction between c-Abl and Abi-1 is important for the regulation of proteins such as p85, a PI3-kinase subunit [33], and CAS [34]. PIP3 has been reported to be necessary for WAVE2 translocation to the leading edge and the formation of Rac-induced protrusions [35]. It is possible that a decreased amount of PIP3 may cause insufficient WAVE2 complex activation and/or membrane recruitment. In either case, our results clearly showed that the NESH/Abi-3-based WAVE2 complex is functionally different from the Abi-1-based one.

It is interesting that the expression of NESH/Abi-3 inhibits migration of SRD cells but not NIH3T3 cells in the wound healing assay. This finding indicates that the mechanism of cell motility is not identical for these two types of cells. Ichigotani et al. reported that expression of NESH/Abi-3 inhibits the motility of human glioblastoma (U87MG) cells as well as SRD cells [17]. A possible difference between these cells and NIH3T3 cells might be the activation of c-Abl kinase. c-Abl can be activated downstream of v-Src [36]. In SRD cells that express v-Src, c-Abl activity may be increased, so that Abi-1 could serve as an adaptor protein to promote c-Abl-mediated phosphorylation of cell motility-related proteins including WAVE2. Because NESH/Abi-3 does not bind to c-Abl, it cannot serve as an adaptor for c-Abl. Accordingly, c-Abl-mediated phosphorylation may not be promoted. By contrast, in NIH3T3 cells, the c-Abl-mediated phosphorylation may not be a major factor controlling cell motility.

The differences between Abi family proteins are also clear as to the invadopodia formation in SRD cells. Matsuda et al. reported that the expression of NESH/Abi-3 increased the metastasis of SRD cells in the presence of imatinib mesylate [18]. Our results suggest that the increase in metastasis they observed may be associated with an increase in the invadopodia formation potential, because the lateral cellular motility of these cells decreased during imatinib treatment. In addition, our results also clarified that this phenomenon is specific to NESH/Abi-3 among the three Abi proteins. Abi-1 is reported to be involved in the regulation of N-WASP/WASP [37–39], and other actin regulatory proteins such as Mena [6] and MIG10 [40]. It is believed that N-WASP/WASP is critical as an actin polymerization regulator for invadopodia formation. The functional interaction of NESH/Abi-3 with these molecules should be examined in the future.

Replacement of Abi-1 with NESH/Abi-3 reduced invadopodia formation. This may be due to invadopodia formation mediated by Abi1/2-WAVE complex but not by the NESH/Abi-3-WAVE complex. However, since it has been reported that the WAVE complex is not implicated in invadopodia formation, it is more likely that expression of NESH/Abi-3 reduces Abi-1 and Abi-2 expression, which normally bind N-WASP and mediate invadopodia formation. NESH/Abi-3 may not bind N-WASP and hence may not replace Abi1/2 in invadopodia formation. Meanwhile, Smith-Pearson et al. disclosed a critical role of Abl kinases in the matrix degradation and cell invasion [27]. In SRD cells, Abi-1 and Abi-2 may regulate c-Abl-mediated phosphorylation, and NESH/Abi-3 may compete with Abi-1 and/or Abi-2 to inhibit the invadopodia formation. However, the above hypotheses alone cannot explain why NESH/Abi-3 promotes the invadopodia formation when c-Abl is inhibited. One possibility is the existence of another pathway downstream of the Src activation, this pathway being negatively regulated by c-Abl. When c-Abl kinase is inhibited, this pathway may be activated, so that in the presence of NESH/Abi-3, this pathway may induce the invadopodia formation. Likewise, a previous study demonstrated that loss of c-Abl facilitates anchorage independence in a cellular context-dependent manner [41]. The existence of a transformation-promoting pathway negatively regulated by c-Abl may be plausible. In addition, a recent study showed that Abl kinase inhibitor treatment promotes invadopodia formation in certain cancer cells [42]. In view of the above, it is clinically important to elucidate the signaling pathway activated in the presence of the Abl kinase inhibitor.

To date, the physiological function of NESH/Abi-3 remains unclear. NESH/Abi-3 is abundant in the spleen and thymus among tissues [21]. In addition to HUVEC, NESH/Abi-3 is expressed in several blood cell lines. In these cells, some WAVE2 complexes should contain NESH/Abi-3, which does not seem to respond to c-Abl. These cells may require two types of WAVE2 complexes to maintain their actin-based cellular cytoskeleton organization. In addition, macrophages or endothelial cells produce podosomes, which are a matrix-degrading ventral cell surface structure similar to invadopodia. NESH/Abi-3 might be involved in the podosome formation in these cells.

In this study, we examined the structural difference between Abi-1 and NESH/Abi-3. The study involving chimeric Abi molecules showed the importance of amino acids 270 to 336 of Abi-1. In the model presented by Dai and Pendergast [2], the SH3 domain of Abi-2 interacts with the proline-rich region of c-Abl, and reciprocally, the most N-terminal proline-rich region of Abi-2 binds to the SH3 domain of c-Abl. Most likely, Abi-1 interacts with c-Abl in a similar manner. Since neither a domain structure nor a motif is present in the a.a. 270–336 region of Abi-1, it is unlikely that this inter-domain region directly interacts with c-Abl. One possible function of this region is to keep the two c-Abl-binding domains of Abi-1 (the most N-terminal proline-rich region and the SH3 domain) at proper positions so that Abi-1 can bind to c-Abl. Because the amino acid length between the most N-terminal proline-rich region and the SH3 domain of NESH/Abi-3 is shorter than that in the case of Abi-1 or Abi-2, NESH/Abi-3 may not be able to maintain the conformation that supports the binding to c-Abl. According to computer modeling, the distance between the most N-terminal proline-rich region and the SH3 domain of NESH/Abi-3 is about 8 Å shorter than that in the case of Abi-1, which supports the above idea. Recently, analysis of the crystal structure of the WAVE complex was reported [43]. In this analysis, an Abi-2 mutant without a C-terminal region including the proline-rich region and the SH3 domain was used. Thus, it should be intriguing to determine what effects are exerted on the structural and functional regulation of the WAVE complex by amino acids 270 to 336 of Abi-1.

## Conclusions

In this study, we examined the function of NESH/Abi-3 and compared it with those of Abi-1 and Abi-2. We showed that the NESH/Abi-3-based WAVE2 complex could not create lamellipodial protrusions in response to FN stimulation. In addition, we showed that expression of NESH/Abi-3, but not Abi-1 or Abi-2, reduced the formation of invadopodia in v-*src*-transformed cells, and increased invadopodia formation when c-Abl was inhibited. We subsequently defined the regions that determine the functional differences between Abi-1 and NESH/Abi-3. Our results suggest that NESH/Abi-3 plays a different role from those of the other two mammalian Abi proteins in the signaling pathway involving c-Abl and possibly c-Src.

## Methods

### Materials

FN and TRITC-phalloidin were purchased from BD Biosciences and Sigma-Aldrich, respectively. The following antibodies were used: anti-Abl (8E9; Pharmingen), anti-FLAG (M2; Sigma-Aldrich), anti-glutathione *S*-transferase (GST) (Santa Cruz Biotechnology), anti-phosphotyrosine (4G10; Upstate Biotechnology), anti-Abi-1 (1G9; MBL Inc.), anti-PIR121/Sra-1 (Upstate Biotechnology), anti-Abi-2 (H-50; Santa Cruz Biotechnology), anti-Arp3 (abcam), anti-Rac (Upstate Biotechnology), anti-cortactin (Millipore), and anti-α-tubulin (Sigma-Aldrich) antibodies. A rabbit anti-WAVE2 antibody was prepared in our laboratory and used for immunoblotting analysis. A rabbit anti-WAVE2 antibody used for immunofluorescence analysis was a kind gift from Drs. T. Takenawa and D. Yamazaki. A rabbit or mouse anti-NESH polyclonal antibody was previously described [21]. An anti-mouse NESH monoclonal antibody was prepared in this study. A cDNA for mouse NESH (AK008928) were obtained from RIKEN. The cDNA fragment encoding 204a.a.–367a.a. of mouse NESH was inserted into an expression vector, pGEX-4T-1 (Amersham Biosciences). The GST-tagged mouse NESH fragment was expressed in *Escherichia coli* cells, purified, and then injected into BALB/c mice. Hybridoma cells producing antibodies against mouse NESH were obtained according to the standard protocol. The obtained monoclonal antibody (2H7) reacts with both mouse and human NESH, but not Abi-1 or Abi-2.

### Cell culture

293T cells were grown in Dulbecco’s modified Eagle’s medium supplemented with 10 % fetal bovine serum and antibiotics. NIH3T3 cells were grown in Dulbecco’s modified Eagle’s medium supplemented with 10 % calf serum (CS) and antibiotics. v-*src*-transformed NIH3T3 cells (SRD cells) were grown in DMEM supplemented with 10 % CS, 1 μg/mL puromycin, and antibiotics.

### Establishment of stable cells

The cDNA for each of FLAG-Abi-1, FLAG-Abi-2, FLAG-NESH, Abi-1, NESH, and chimeric proteins N-1 and N-5 was subcloned into the pCX4bsr vector. To prepare a recombinant retrovirus, each plasmid was co-transfected together with pGP Vector and pE-eco Vector (Takara) into PLAT-E cells using Fugene 6 (Roche). At 24 h after the transfection, the medium was changed to fresh. After an additional 24 h, the supernatant was collected and passed through a filter of 0.22-μm pore size. The resulting virus solution was added to NIH3T3 cells. After 24 h incubation, the medium was changed to fresh containing 10 μg/mL blasticidin to establish stable cells expressing each Abi protein. A mixed population of drug-resistant cells was used for experiments. The empty vector was used to establish control cells. The mixed population of drug-resistant cells was cultured in DMEM supplemented with 10 % CS, 10 μg/mL blasticidin, and antibiotics.

A recombinant retrovirus containing *v-src* was produced with the pBabe Puro *v-src* vector (a kind gift from T. Akagi), and NIH3T3 cells were infected with the virus to produce SRD cells. The cDNA for Abi-1, FLAG-Abi-2, or NESH was subcloned into the pCX4neo vector to produce the pCX4neo-Abi-1, pCX4neo-FLAG-Abi-2, or pCX4neo-NESH plasmid. Virus solutions were obtained as described above. Stable SRD cells expressing Abi-1, FLAG-Abi-2, or NESH were obtained in the same manner except that 1.6 mg/mL G418 and 1 μg/mL puromycin were used for selection. A mixed population of drug-resistant cells was used. The cells were maintained in the presence of 1.6 mg/mL G418 and 1 μg/mL puromycin.

### Cell-spreading assay

Confluent cells were detached by trypsinization and held in suspension for 45 min. The resulting cells were plated onto FN-coated coverslips and then incubated at 37 °C for the indicated times. Subsequently, the cells were fixed, washed gently with phosphate-buffered saline, and then subjected to immunofluorescence analysis.

### Rac-GTPase assay

The CRIB domain of PAK1 (a.a. 67–150) was amplified by means of a PCR using a human leukocyte cDNA library as a template. The cDNA encoding the PAK-CRIB was subcloned into the pGEX-2T vector. Expression and purification of GST-CRIB were performed in accordance with the manufacturer’s instructions. Then, 250 μg aliquots of cellular lysates were incubated for 90 min at 4 °C with the GST-CRIB immobilized on glutathione beads.

### Wound-healing assay

NIH3T3 cells or SRD cells expressing each of the Abi proteins were plated on FN-coated coverslips while being kept confluent. The cells were wounded by manual scraping with a yellow tip. At 0 and 12 h after the scraping, differential interference contrast images were obtained and the wounded area in each image was measured using the ImageJ software.

### Invadopodia assay

TRITC gelatin-coated dishes were prepared as described previously [44]. Cells were cultured on the gelatin matrix for 7 h, fixed with paraformaldehyde, and then stained with an anti-cortactin antibody. To assess matrix degradation, images were obtained by confocal microscopy. The gelatin degradation area exhibiting overlapping cortactin staining was quantitated with the ImageJ software.

### Imatinib treatment

Cells were incubated in a 5 μM or 10 μM imatinib-containing medium for 24 h at 37 °C prior to assays. Assays were performed in the presence of the same concentrations of imatinib.

### RNA interference

Four siRNAs against mouse c-Abl were obtained from QIAGEN. After checking their knockdown efficiency, Mm_Abl1_2 was selected and used for the experiments. Control SRD cells and SRD cells expressing each of the Abi proteins were transfected with Mm_Abl1_2 at a final concentration of 100 nM using Lipofectamine RNAiMAX (Life Technologies) according to the manufacturer’s protocol. At 72 h after the transfection, the cells were plated onto TRITC gelatin-coated dishes and analyzed as described above.

### Expression plasmids

The cDNAs for human Abi-1 and human NESH were described previously [6, 21]. The interactor protein AblBP4 [Homo sapiens] GenBank: AAD00897.1 (451 a.a.) was hereafter used as the cDNA for Abi-1. This Abi-1 clone corresponds to isoform 3 described by Dubielecka et al. [45]. A cDNA for human Abi-2 was amplified by means of a PCR using a human leukocyte cDNA library (CLONTECH). The obtained cDNA was found to be identical to that for abl interactor 2 (accession number BC001439). A cDNA for human WAVE2 was a kind gift from Dr. Takenawa. pcDNA3 c-Abl was a kind gift from Dr. David Baltimore. Mammalian expression plasmids, pEBG (a gift from Dr. Bruce Mayer) and pFLAG-CMV-6 (Sigma-Aldrich), were used to express proteins fused with an N-terminal GST and FLAG tag, respectively. Chimeric mutants between Abi-1 and NESH were produced by PCR and verified by DNA sequencing. Abi-1/NESH•SH3 was composed of the N-terminal 391 amino acids (a.a.) of Abi-1 and the SH3 domain of NESH (a.a. 311–366). NESH/Abi-1•SH3 was composed of the N-terminal 310 a.a. of NESH and the SH3 domain of Abi-1 (a.a. 392–451). Chimeric proteins N-1, N-2, N-3, N-4, and N-5 were fusion proteins comprising a.a. 1–168, 1–180, 1–199, 1–239, and 1–263 of NESH, and a.a. 174–451, 191–451, 213–451, 270–451, and 336–451 of Abi-1, respectively. The NESH chimera containing a.a. 270–336 of Abi-1 was created by inserting a.a. 270–336 of Abi-1 between proline 236 and leucine 237 of NESH/Abi-3. Abi-1 Y213F was created by replacing tyrosine 213 of Abi-1 by a phenylalanine.

### *In Vitro* binding assay

GST-tagged and FLAG-tagged proteins were expressed in 293T cells, and pull-down experiments were performed as described previously [6].

### Phosphorylation analysis

Phosphorylation analysis was performed as described previously [6]. Briefly, 293T cells were transfected with expression plasmids using LipofectAMINE PLUS reagent (Invitrogen). At 24 h after the transfection, cells were lysed in a lysis buffer containing phosphatase inhibitors (1 mM sodium orthovanadate and 10 mM sodium fluoride), and then analyzed by Western blotting with an anti-phosphotyrosine antibody. In some experiments, lysates were pulled down and the bound proteins were analyzed by Western blotting.

### Immunoprecipitation

Immunoprecipitation was performed essentially as described previously [21]. Briefly, lysates were incubated with the respective antibodies overnight at 4 °C while rocking. Then, protein G beads were added, incubated, centrifuged, and washed. The immunoprecipitated proteins were then boiled in a sample buffer, subjected to SDS-PAGE, and transferred onto an Immobilon-P membrane.

### Prediction of the three-dimensional structures of Abi-1 and NESH/Abi-3

The three-dimensional structure of the full-length Abi-1 was calculated using the Robetta server [46] in accordance with the Rosetta *de novo* protocol [47, 48]. Using this structure as a template, the three-dimensional structure of NESH/Abi-3 was predicted with the Modeller [49] using a homology modeling protocol. All the calculations for the prediction of the structures were carried out under default parameter settings.

## Additional file

Additional file 1: Figure S1.
**(A)** One hundred μg of lysates prepared from NIH 3T3 cells and mouse brain were analyzed by Western blotting with the indicated antibodies. For Abi-2, two different dilutions of the antibody were used to obtain the results. Several Abi-1 or Abi-2 bands observed for the mouse brain lysates may reflect splicing variants, or the phosphorylation or partial degradation of these proteins. **(B)** Control NIH3T3 cells and NIH3T3 cells expressing Abi-1 or NESH were plated onto FN-coated coverslips, and analyzed as described in Fig. 1a. **(C)** Quantitative analysis of the cells in (B). Cells with lamellipodial structures were counted under a fluorescence microscope. At least 100 cells were analyzed for each sample. Data represent the means ± S.D. for three independent experiments. **(D)** The wound-healing assay was performed using the control NIH3T3 cells and NIH3T3 cells expressing Abi-1 or NESH, and analyzed as described in Fig. 1e. **(E)** Mouse brain lysates were immunoprecipitated with a mouse IgG, the anti-Abi-1 monoclonal antibody (1G9), or the anti-NESH monoclonal antibody (2H7). The precipitated proteins were analyzed by Western blotting with the indicated antibodies. **(F)** Rac activity assay. Cell lysates of the control and FLAG-NESH expressing NIH3T3 cells were incubated with GST-CRIB immobilized on glutathione beads, and then the precipitated proteins were analyzed by Western blotting with an anti-Rac antibody. To determine the total amount of Rac, 4 % of each lysate was analyzed (Input). (TIFF 19164 kb)

## Abbreviations

SH3: Src homology 3
GST: Glutathione *S*-transferase
a.a.: Amino acids
FN: Fibronectin
HUVEC: Human umbilical vein endothelial cells
