# Supplementary figures and images for: The NESH/Abi-3-based WAVE2 complex is functionally distinct from the Abi-1-based WAVE2 complex
Source: Cell Commun Signal. 2015 Oct 1;13:41. doi: 10.1186/s12964-015-0119-5 (PMC4589964; doi:10.1186/s12964-015-0119-5)

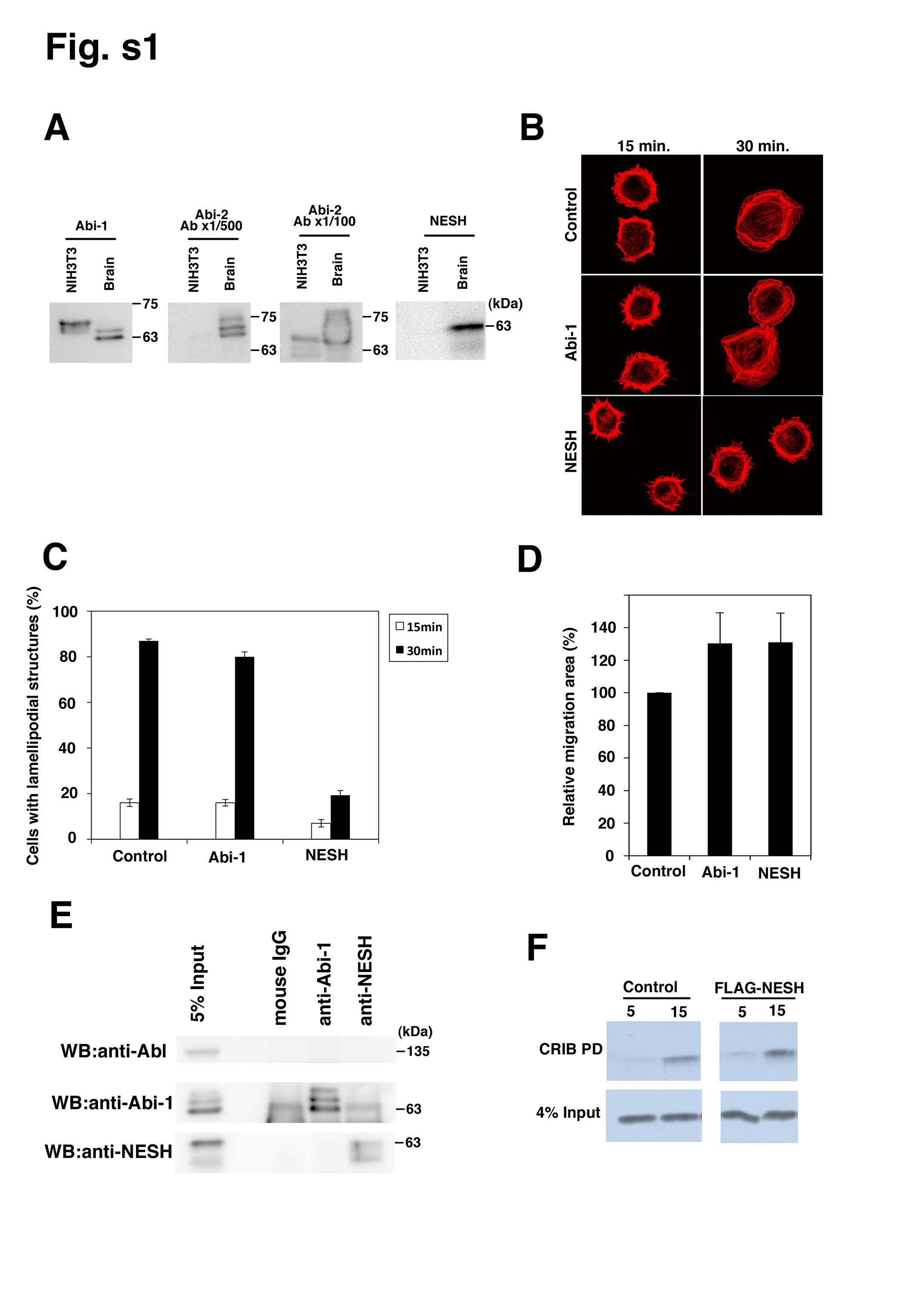

Supplement: Additional file 1: Figure S1. — (A) One hundred μg of lysates prepared from NIH 3T3 cells and mouse brain were analyzed by Western blotting with the indicated antibodies. For Abi-2, two different dilutions of the antibody were used to obtain the results. Several Abi-1 or Abi-2 bands observed for the mouse brain lysates may reflect splicing variants, or the phosphorylation or partial degradation of these proteins. (B) Control NIH3T3 cells and NIH3T3 cells expressing Abi-1 or NESH were plated onto FN-coated coverslips, and analyzed as described in Fig. 1a. (C) Quantitative analysis of the cells in (B). Cells with lamellipodial structures were counted under a fluorescence microscope. At least 100 cells were analyzed for each sample. Data represent the means ± S.D. for three independent experiments. (D) The wound-healing assay was performed using the control NIH3T3 cells and NIH3T3 cells expressing Abi-1 or NESH, and analyzed as described in Fig. 1e. (E) Mouse brain lysates were immunoprecipitated with a mouse IgG, the anti-Abi-1 monoclonal antibody (1G9), or the anti-NESH monoclonal antibody (2H7). The precipitated proteins were analyzed by Western blotting with the indicated antibodies. (F) Rac activity assay. Cell lysates of the control and FLAG-NESH expressing NIH3T3 cells were incubated with GST-CRIB immobilized on glutathione beads, and then the precipitated proteins were analyzed by Western blotting with an anti-Rac antibody. To determine the total amount of Rac, 4 % of each lysate was analyzed (Input). (TIFF 19164 kb) [file 12964_2015_119_MOESM1_ESM.tiff]
